# Recent Advances in Non-Enzymatic Glucose Sensors Based on Metal and Metal Oxide Nanostructures for Diabetes Management- A Review

**DOI:** 10.3389/fchem.2021.748957

**Published:** 2021-09-22

**Authors:** Gowhar A. Naikoo, Hiba Salim, Israr U. Hassan, Tasbiha Awan, Fareeha Arshad, Mona Z. Pedram, Waqar Ahmed, Ahsanulhaq Qurashi

**Affiliations:** ^1^ Department of Mathematics and Sciences, College of Arts and Applied Sciences, Dhofar University, Salalah, Oman; ^2^ College of Engineering, Dhofar University, Salalah, Oman; ^3^ Department of Biochemistry, Aligarh Muslim University, Aligarh, India; ^4^ Mechanical Engineering-Energy Division, K. N. Toosi University of Technology, Tehran, Iran; ^5^ School of Mathematics and Physics, College of Science, University of Lincoln, Lincoln, United Kingdom; ^6^ Department of Chemistry, Khalifa University of Science and Technology, Abu Dhabi, United Arab Emirates

**Keywords:** metal and metal oxide nanostructures, non-enzymatic glucose sensors, diabetes, early detection, mechanism, challenges, possible solutions

## Abstract

There is an undeniable growing number of diabetes cases worldwide that have received widespread global attention by many pharmaceutical and clinical industries to develop better functioning glucose sensing devices. This has called for an unprecedented demand to develop highly efficient, stable, selective, and sensitive non-enzymatic glucose sensors (NEGS). Interestingly, many novel materials have shown the promising potential of directly detecting glucose in the blood and fluids. This review exclusively encompasses the electrochemical detection of glucose and its mechanism based on various metal-based materials such as cobalt (Co), nickel (Ni), zinc (Zn), copper (Cu), iron (Fe), manganese (Mn), titanium (Ti), iridium (Ir), and rhodium (Rh). Multiple aspects of these metals and their oxides were explored vis-à-vis their performance in glucose detection. The direct glucose oxidation *via* metallic redox centres is explained by the chemisorption model and the incipient hydrous oxide/adatom mediator (IHOAM) model. The glucose electrooxidation reactions on the electrode surface were elucidated by equations. Furthermore, it was explored that an effective detection of glucose depends on the aspect ratio, surface morphology, active sites, structures, and catalytic activity of nanomaterials, which plays an indispensable role in designing efficient NEGS. The challenges and possible solutions for advancing NEGS have been summarized.

## Introduction

**FIGURE 1:**
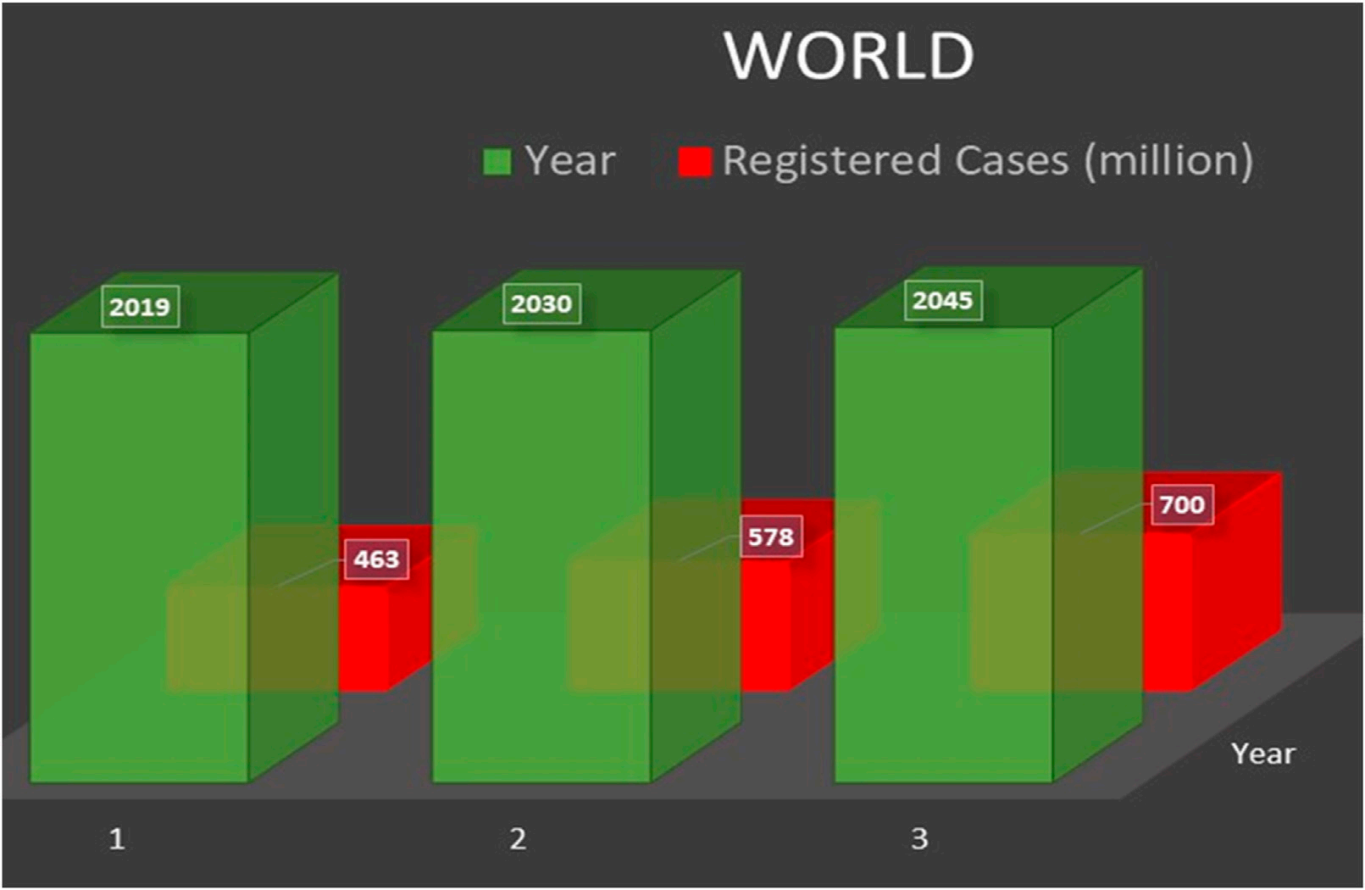
Estimated and projected cases of diabetics across the world (IDF, 2019).

Diabetes, a chronic condition, is considered one of the deadliest and most rambling diseases globally. The latest report of the International Diabetes Federation (IDF), Atlas, declared that 463 million adults (20–79 years) lived with diabetes and projected that this increase might reach up to 700 million by 2045. Among the top ten countries with the highest number of diabetic patients (age 20–79 years), China is leading the list, followed by India. Figure 1 reveals the estimated in 2019 and projected cases of diabetics across the world in 2030 and 2045 (IDF, 2019) and describes the possible increase in diabetes (by %) in 2019, 2030, and 2045, respectively, in different regions across the globe (IDF, 2019). It has been concluded that Europe is predicted to have the lowest increase in diabetes (15%). In comparison, the Middle East and North Africa (MENA) are expected to have a predominant rise in people with diabetes (96%) (IDF, 2019). To overcome this challenging increase in diabetes, the scientific community needs to make enormous efforts to develop highly efficient, easily accessible, stable NEG sensors to monitor the glucose level at the early stages of diabetes (Teymourian et al., 2020).

Over the past couple of decades, scientists have consistently fabricated advanced nanostructured materials to develop glucose sensors with high sensitivity and selectivity (Wang et al., 2013; Hwang et al., 2018). However, these advanced materials are mostly inorganic nanoparticles (NPs) (Hwang et al., 2018), nanosheets (Joshna et al., 2020), and nanowires (Thangasamy et al., 2020), which makes it possible to tailor the functionality and surface structure of the resulting materials (Salihoglu and Kahlout, 2019) and play a dynamic role in the expansion of electrochemical sensors for glucose detection. These sensors are categorised into two groups: enzymatic glucose (EG) and NEG sensors (Jayram et al., 2016). EG sensors possess high specific catalytic action and efficiency, besides their excellent selectivity, sensitivity, and mild measurement conditions (Bruen et al., 2017). However, EG sensors are significantly influenced by different environmental conditions such as pH, temperature, toxic chemicals, and humidity, which has limited their use (Numan et al., 2017; Tee et al., 2017) and led to NEG sensors’ production (Zhang and Liu, 2017). Consequently, NEG sensors have received much attention in recent years due to their fast and precise response, low cost, and excellent sensitivity (Wu et al., 2019). In particular, considerable consideration has been put forward to fabricate highly efficient materials to improve sensors’ performance by tailoring their shape, size, composition, adsorption capacity, electron transfer properties, and specific surface area. Over the past couple of decades, various metal and metal oxide materials have been employed for glucose sensing applications (Toghill and Compton, 2010; Guler and Dilmac, 2019; Hossain and Slaughter, 2020; Hassan et al., 2021)–(Toghill and Compton, 2010; Guler and Dilmac, 2019; Hossain and Slaughter, 2020; Hassan et al., 2021). Nanomaterials of noble metals (Ag, Au, Pd, and Pt) (Wang et al., 2014; Baghayeri et al., 2018a; Ma et al., 2019) were considered excellent choices for constructing NEG sensors because of their high efficiency in glucose electrooxidation. However, the unaffordable cost of these metals for the development of NEG sensors has limited their use. Consequently, researchers have commenced designing NEG sensors based on metals and their oxides, and in particular, the focus was given to Ni (Rajendran et al., 2018), Zn (Yang et al., 2016a; Ognjanović et al., 2019), Cu (Shabnam et al., 2017), NiO (Baghayeri et al., 2018b), CuO (Shabnam et al., 2017), NiCo_2_O_4_ (Baghayeri et al., 2018b; Rajendran et al., 2018), Fe (Li et al., 2015; Marie et al., 2018), Mn (Li et al., 2015; Xie et al., 2018a), Ti (AL-Mokaram et al., 2017), Ir (Dong et al., 2018a; Dong et al., 2019), and Rh (Dong et al., 2018b) etc.

Co metal ions based on phosphides (Tian et al., 2015), phosphates (Theerthagiri et al., 2017), oxides (Vilian et al., 2018), and nitrites (Xie et al., 2018b) have exhibited promise for their employment in electrochemical sensing (Han et al., 2015). However, due to the semiconducting properties of Co-based hydroxides and oxides, their electrocatalytic capabilities have decreased severely. Therefore, previous studies have paid attention to improving the electro-conductivity of Co-based hybrid catalysts (Wang et al., 2016a). In addition to this, nickel-based materials were also considered a potential choice for synthesizing electrochemical NEG sensors. Especially, NiO (Ma et al., 2018), Ni/Al layered double hydroxide (Kumar et al., 2018), Ni(OH)_2_ (Yang et al., 2016b), Ni metal (Sun et al., 2020), and Ni(II)-based metal-organic coordination polymer (Zhe et al., 2019) all showed better electrochemical activity for glucose oxidation and hence have been employed in fabricating enzyme-free glucose sensors. Similarly, zinc oxide is one of the most multifunctional and significant electrode material candidate because of its unique physical, chemical, mechanical and electrochemical properties, which is evident because of the electron features, wide bandgap (Hussain et al., 2013), biocompatibility (Nain et al., 2020), cost-effective synthesis (Narayana et al., 2020), optical transparency (Kulkarni et al., 2015), comfortable and better electrochemical performance (Ridhuan et al., 2018; Pradeeswari et al., 2019).

Copper oxide (CuO) based materials display semiconducting properties along with unusual electronic and optical features (Piri et al., 2019). At the nano level, CuO exhibit better catalytic activity when compared to CuO as a whole (in the bulk form). Various studies have shown the electrochemical detecting properties of CuO and its derivatives (Yuan et al., 2017; Avinash et al., 2019). These oxides boost the sensing ability of the electrodes in the sensor and display lowered LOD. Also, elements like Fe possesses magnetic properties. Their nanoparticles (NPs) have magnetic properties, are biocompatible, and less toxic; therefore can be used in developing biosensors to analyze biomolecules (Reetz et al., 1998; Li et al., 2003). Likewise, numerous studies on manganese oxide-based sensors for glucose detection (Yang and Hu, 2010; Si et al., 2013; Wang et al., 2015; Liu et al., 2016a) have shown their potential in glucose detection (Yang and Hu, 2010). Elements like Ir (Dong et al., 2019) and Rh (Dong et al., 2018b) and their oxides have also shown favorable properties for glucose detection in NEGS. These materials, therefore, are promising potential substitutes for developing highly efficient, reliable and stable NEG sensors for early detection of glucose in diabetic patients.

## Electrochemical Detection of Glucose

Glucose detection with low cost, accurate and fast processes is vital for food engineering, pharmaceutical analysis, environmental monitoring, and clinical biochemistry (Wang et al., 2017; Hosu et al., 2019). Many detection methods for glucose sensing have been discovered, such as calorimetry, electrochemistry, conductometry, fluorescent spectroscopy, and optical rotation (Lv et al., 2016). These analytical methods typically address various issues, such as tedious detection procedures, interaction with coexisting anionic or cationic organisms, long assay times, and high-cost equipment (Gao et al., 2016). Hence, most electrochemical glucose sensors depend on electrochemical methods due to their portability, selectivity, and simplicity. Furthermore, they display pre-eminent stability, fast response time, less cost, and low LOD (Mohd Yazid et al., 2014; Sehit and Altintas, 2020).

### Direct Glucose Oxidation *via* Metallic Redox Centers

#### Chemisorption Model

**FIGURE 2:**
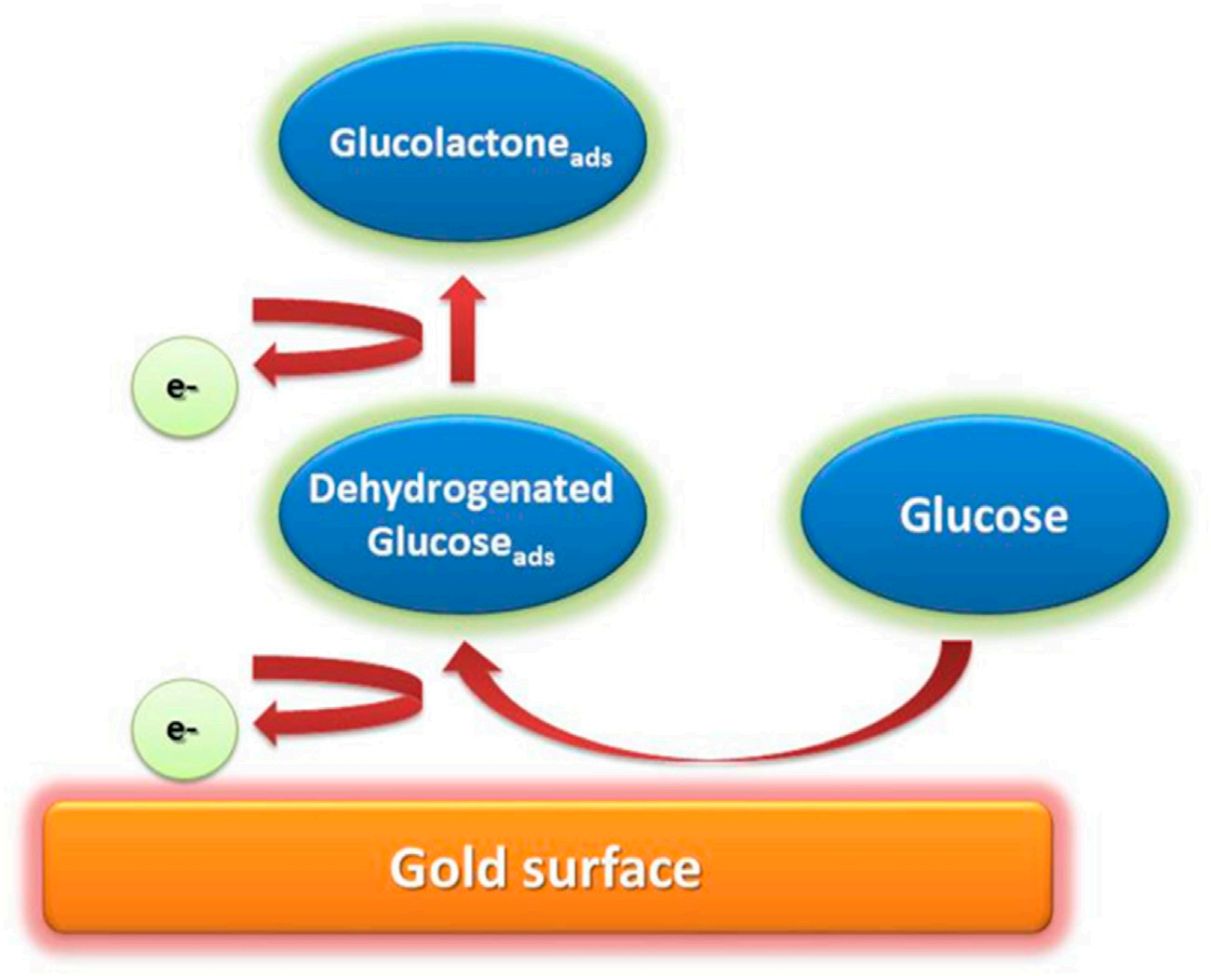
Mechanism of glucose oxidation in Chemisorption Model.

Most electrocatalytic processes take place *via* the adsorption of reactant molecules to active electrode sites. Adsorption of the reactant molecules is accompanied by breaking bonds and new intermediate formation (Abunahla et al., 2019). The adsorption mechanism is affected by different factors such as non-metal catalysts deficiencies, unoccupied d-orbitals at transition metal (TM) centres, and the redox center’s optimal electronic state (Niu et al., 2016). The interaction between the electrode and product decreases when the redox center’s oxidation state changes, resulting in the reaction products desorption from the electrode’s surface (Figure 2). A method that involves the adsorption and desorption of reactants on the electrode is referred to as chemisorption model (Toghill and Compton, 2010; Seh et al., 2017). In this model, the electrode surface’s chemical interaction with C-1 of a glucose molecule and its hydrogen atom upsurges as the glucose molecule reaches the electrode, which causes C-1 to dehydrogenate and adsorb to the electrode surface. Subsequently, when electrooxidation of adsorbents occurs, gluconolactone is oxidized to gluconic acid *via* several pH-dependent reaction routes (de Mele et al., 1982; Beden et al., 1996; Li et al., 2018; Xia and Guo, 2019).

#### IHOAM Model

**FIGURE 3:**
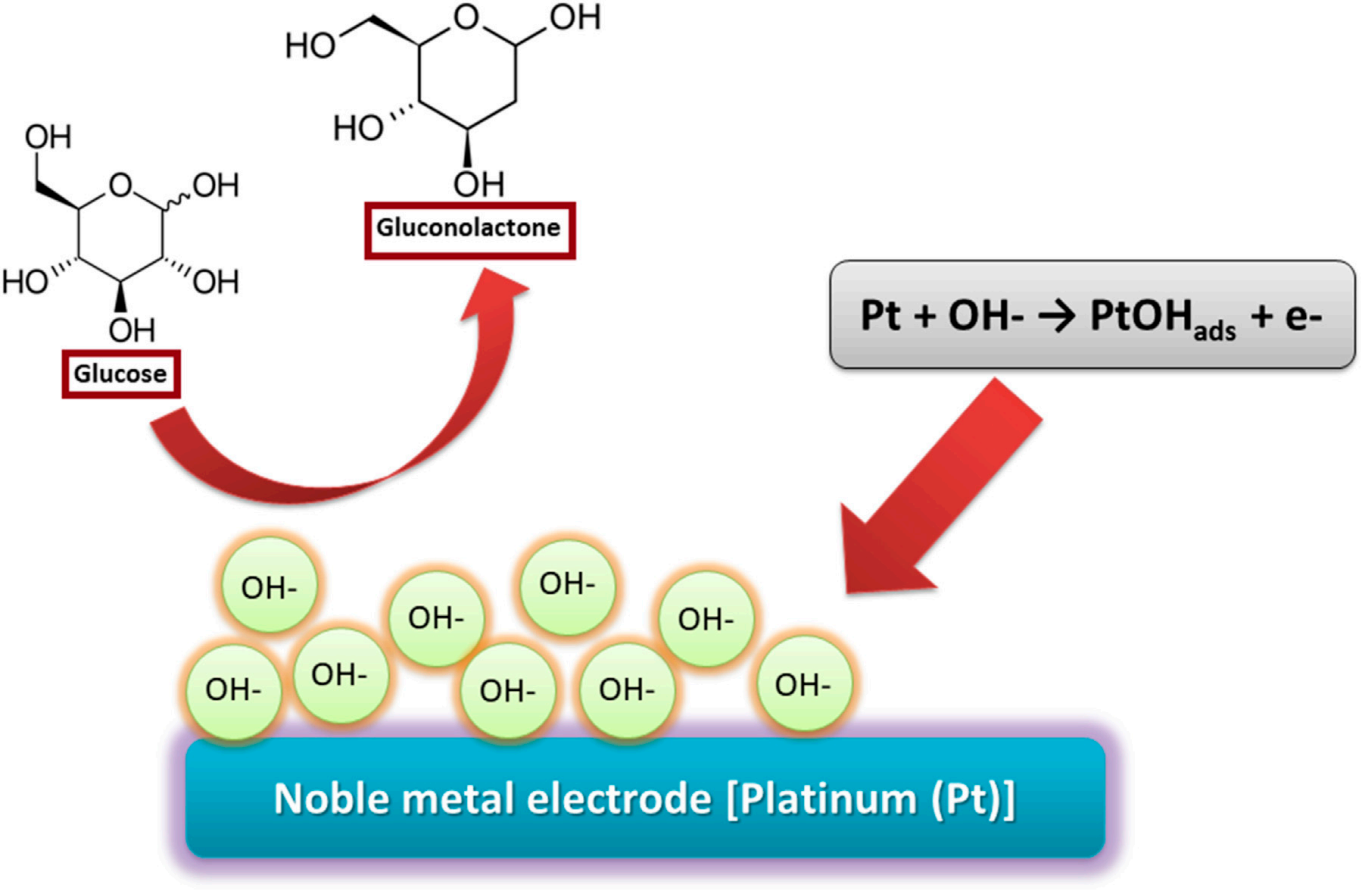
Mechanism of glucose oxidation in IHOAM Model.

IHOAM model was suggested by Burke et al., which involves reactive hydroxide species on the electrode surface (OH_ads_) produced during the electrocatalysis and their effect on many organic molecules’ redox reactions (Figure 3). Direct oxidation of the reactants takes place by hydroxyl radicals. Simultaneously, reactive OH_ads_ pre-monolayer with a low lattice coordination value is created on the electrode surface and mediates different redox reactions (Tian et al., 2019). Various studies on using other metal electrodes (Me) for glucose oxidation proved the participation of the reactive OH_ads_ (Burke and Ryan, 1992; Lertanantawong et al., 2008). Since IHOAM and chemisorption models ultimately presume noble metal electrodes, for example, Au and Pt, many metal oxide-based electrodes are not entirely related to this explanation. Hence, TM centers’ redox reaction can clarify the oxidation of glucose for materials like Co(Cataldi et al., 1995; Chang et al., 2014) and Ni (Morales et al., 2018; Juodkazytė et al., 2019). Researchers have revealed that direct oxidation of glucose is affected by different reaction conditions, where it is rarely acidic and usually alkaline or neutral. In an alkaline medium, reactive OH_ads_ are formed, while in an acidic medium, metal oxide electrode materials are unstable. However, the domination of readily oxidizable β-glucopyranoses occurs at greater pH due to mutarotation (Cheng et al., 2001; Sehit et al., 2020; Sun et al., 2021)–(Cheng et al., 2001; Sehit et al., 2020; Sun et al., 2021).

## Electrochemical Detection of Glucose on Co, Ni, Zn, Cu, Fe, Mn, Ti, Ir, Rh, Pt, Pd, Au BASED NEGS

### Cobalt-Based NEGS

The application of cobalt and its oxides in sensor technology has significant advantages that include large bandgap, biological compatibility, low cost, and high stability (Soomro et al., 2015; Gong et al., 2019). Owing to its good selectivity and reproducibility, various studies have used cobalt and its oxides to develop NEG sensors in recent years (Soomro et al., 2015; Tian et al., 2018; Janyasupab and Promptmas, 2019; Li et al., 2019; Strakosas et al., 2019; Wang et al., 2019; Zhao et al., 2020). One of the earliest works on cobalt oxide (Co_3_O_4_) based sensors is by Ding et al. (2010). They developed electrospun nanofibers displaying a rapid response rate, high sensitivity of 36.25 μA mM^−1^ cm^−2^ and increased reproducibility. The sensor also showed high selectivity against ascorbic and uric acids with a LOD of 0.97 μM. Though this sensor showed an agreement with results using a commercial glucose sensor, this sensor displayed a maximum activity under alkaline conditions. In another similar study by Soomro et al. (2015), Co_3_O_4_ nanostructures were used to develop NEGS that indicated a good sensitivity of 27.33 μA mM^−1^ cm^−2^ and high stability. The authors recorded a wide linear range of 0.5–5.0 mM and a LOD of 0.8 μM. In addition, the sensor showed a high sensitivity for glucose against ascorbic and uric acids and dopamine. However, like the study by their contemporaries, this sensor too showed maximum activity under alkaline conditions. Likewise, in another closely similar recent study, Co_3_O_4_ nanostructures displayed superior sensitivity of 839.3 μA mM^−1^ cm^−2^ and high stability for non-enzymatic glucose detection (Tian et al., 2018). In a study by Li et al. (2019), similar observations were recorded. The authors developed a Co-Ni hydroxide-based sensor with a high sensitivity of 1911.5 μA mM^−1^ cm^−2^ and a LOD of 0.127 μM. In addition, the sensor had a wide linear range of 0.00025–1 mM and 1–5 mM; and demonstrated very high stability and remarkable selectivity under alkaline conditions.

**FIGURE 4:**
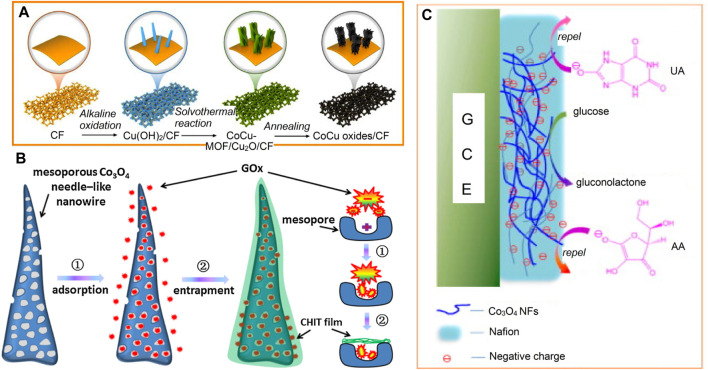
Cobalt-based NEGS **(A)** Schematic illustration of the fabrication process of CoCu oxides/CF. Adapted with permission from ref. (Wei et al., 2020), copyright@2020 (Elsevier). **(B)** Schematic illustration of the mesoporous needle-like Co_3_O_4_ nanowires for immobilizing the redox enzyme GOx by adsorption and entrapment. Adapted with permission from ref. (Ding et al., 2016), copyright@2016 (Elsevier). **(C)** Schematic illustration for the selective catalytic reaction towards glucose. Adapted with permission from ref. (Ding et al., 2010), copyright@2010 (Elsevier).

Figure 4 below discusses these various steps involved in the fabrication and the development of cobalt-based NEGS.

**FIGURE 5:**
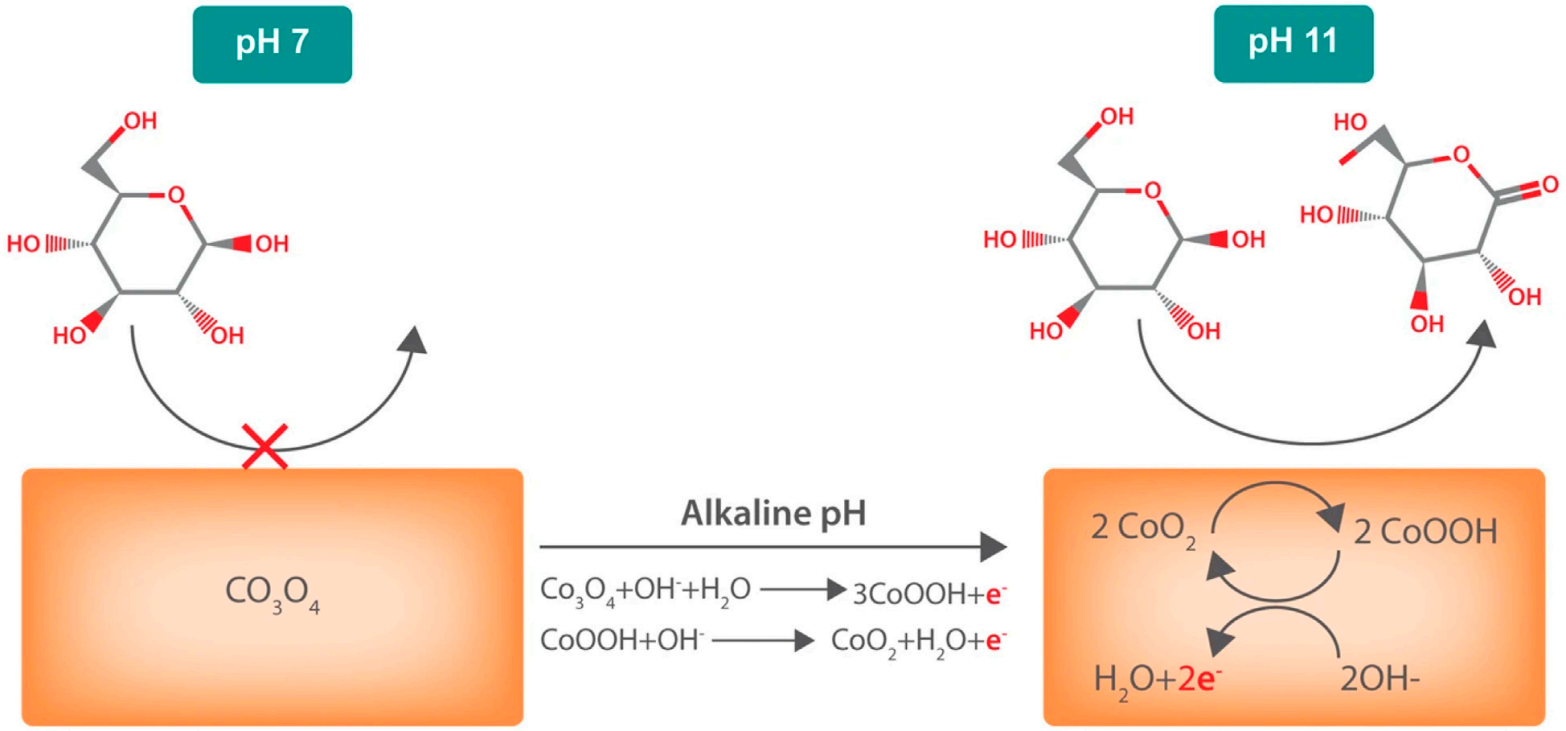
A schematic representation of the glucose sensor operation that shows the sensing mechanism of Co_3_O_4_ contacts. At pH 7, the contact is primarily Co_3_O_4_, which does not oxidize glucose. In alkaline conditions (pH ≥ 11), the contact is now mainly CoO_2_. CoO_2_ species react with glucose and are converted to CoOOH. This CoOOH is then oxidized back to CoO_2_. For every oxidized glucose molecule, the contact collects two electrons measured as I_g_. Reproduced with permission from ref. (Strakosas et al., 2019), copyright@2019 (Nature).

One common theme among the above-discussed sensors is the requirement of hydroxide ions for proper functioning and stability. This particular drawback hinders their adequate application in the determination of glucose levels from biological fluids, including blood, sweat, and tears that have a neutral (4–7) pH range (Strakosas et al., 2019). To overcome this, Strakosas et al. (2019) developed another cobalt oxide-based sensing device that could function and detect glucose molecules under neutral pH conditions. In addition to the fundamental elements of a biosensor, the authors attached a bioelectronic pH control on the sensor surface to regulate the pH of the sensor. Thus, the sensor could induce changes in pH using a Pd contact that causes the absorption of H^+^ from the neutral fluid, causing an enhancement in pH. This thus allowed glucose sensing in biological fluids even at a high pH condition. The glucose sensing mechanism as demonstrated by the authors has been mentioned in Figure 5.

The interfacial reactions occurring at the Co_3_O_4_ glassy carbon electrode *via* the sensing phase is explained in the equations below. As in the first equation, OH^‾^ is a prerequisite for CoO_2_ formation. Hence, the purpose of using NaOH is to form a CoO_2_ oxidant for electrochemical glucose sensing. This is why most studies, as discussed above mentioned the applicability of cobalt-based sensors under alkaline conditions.

$${\text{CoOOH}+\text{OH}}^{-}↔{\text{CoO}}_{2}{+\text{H}}_{2}{\text{O}+e}^{-}$$

$${2\text{CoO}}_{2}{+\text{C}}_{6}{\text{H}}_{12}{\text{O}}_{6}\left(\text{glucose}\right)↔{2\text{CoOOH }+\text{ C}}_{6}{\text{H}}_{10}{\text{O}}_{6}\;\left(\text{gluconolactone}\right)\text{.}$$

Cobalt phosphate nanostructures have also been proposed to electrochemically oxidize from glucose to gluconolactone, as described below (Loeb, 1909).

$${2\text{Co}}^{3+}\to {2\text{Co}}^{4+}{+2e}^{-}$$

$${2\text{Co}}^{4+}{+\text{C}}_{6}{\text{H}}_{12}{\text{O}}_{6}\left(\text{glucose}\right)\to {\;2\text{Co}}^{3+}{+\text{C}}_{6}{\text{H}}_{10}{\text{O}}_{6}\left(\text{gluconolactone}\right){+2\text{H}}^{2+}$$

The linear range, sensitivity, LOD, and response time of various electrode materials based on Co are explored and discussed in Table 1.

**TABLE 1:** Electrochemical detection of glucose on Co, Ni, Zn, Cu, Fe, Mn, Ti, Ir, Rh, Pt, Pd, Au based NEGS.

Electrode material	Linear range (mM)	Sensitivity (μAmM^−1^ cm^−2^)	Limit of detection (μM)	Response time (s)	Ref
Co_3_O_4_ NPs/GCE	0.005–0.8	520.7	0.13	—	Hou et al. (2012)
CoOOH nanosheets	Up to 0.5	967	10.9	—	Lee et al. (2012)
Co-Ni hydroxide nanostructures	0.00025–1	1911.5	0.127	—	Li et al. (2019)
Zn-Co-S BHS	0.005–0.1	2,734.4	2.98	20	Chang et al. (2020)
Co_3_O_4_ nanostructures	0.5–4.5	27.3	0.8	—	Soomro et al. (2015)
CoNiCu Alloy	0.05–1.551	791	0.5	—	Gong et al. (2019)
Co(OH)_2_/3D–graphene foam	0.1–10	3690	0.016	—	Shackery et al. (2016)
CoNiCu Alloy	1.551–4.050	322	0.5	—	Gong et al. (2019)
CuCo carbon nanofibers	0.02–1.1	507	1	—	Li et al. (2015)
Co-Ni nanorods	0.1–1	544	—	—	Vilana et al. (2015)
Co_4_N nanosheets	0.6–10.0	1,137.2	0.1	1.7	Liu et al. (2018a)
Cobalt phosphate nanostructures	1–30	0.0079	0.3	—	Tomanin et al. (2018)
CoP nanorods/GCE	0–5.5	116.8	9	—	Sun et al. (2016)
Ni(OH)_2_-NND	0.02–1 and 1–9	3.20 and 1.41	1.2	—	Ko et al. (2013)
Ni/NiO	0.0005–9	4,400	0.007	—	Singer et al. (2020)
Ni/PANI	0–7	76.8	10	—	Wang et al. (2021)
PtNi NPs/graphene	0.5–15	24.03	16	—	Li et al. (2020)
Ni-C composite	0.02–0.5	670	8	—	Marini et al. (2018)
NiO@SiNPs	Wide linear range	445	0.08	—	Naikoo and Din Sheikh, (2019)
NiO HPA/GCE	0.0025–1.10	1,323	0.32	—	He et al. (2018)
Ni(OH)_2_/Ni foam	0.0000025–0.00105	2,617.4	2.5	—	Xia et al. (2017)
Ni_5_P_4_/GCE	0.002–5.3	149.6	0.7	—	Xiao et al. (2020)
ZnO nanorod	0.1–10	2.97	1,000	—	Chung et al. (2017)
ZnO-CuO NRs/FTO	0.001–8.45	2,961.8	0.40	<2	Ahmad et al. (2017a)
ZnO NPs/GCE	1–8.6	631.30	0.043	<4	Ahmad et al. (2017a)
ZnO nanorods	0.1–13.8	2.97	1,000	—	Chung et al. (2017)
MWCNT/ZnO QDs	0.0001–0.0025	9.36	0.208	<3 s	Vinoth et al. (2021)
Cu-CuO NWs/GCE	0.1–12	122.73	0.05	—	Wang et al. (2010)
PEDOT: PSS-CuO-MWCNTs/PGE	Up to 10	663.2	0.23	—	Amirzadeh et al. (2018)
Cu_2_O-Zn	0.02–1	441.2	0.13	<3 s	Manna et al. (2020)
Ppy–CS–Fe_3_O_4_NP/ITO	1–16	12	234	<3 s	Abdul Amir AL-Mokaram et al. (2016)
Fe_2_O_3_-ZNRs	<18	—	∼12	—	Ahmad et al. (2017b)
MnO_2_/MWNTs nanocomposite	<28	33.19	—	—	Chen et al. (2008)
MnO_2_/graphene composite	0.04–2	3.3	10	—	Liu et al. (2016a)
Ppy-CS-TiO₂	1–14	0.008	614	<3 s	AL-Mokaram et al. (2017)
IrO_2_ NFs	—	22.22	2.9	—	Dong et al. (2018a)
Rh_2_O_3_ NCs	—	11.46	3.1	—	Dong et al. (2018b)
PtNFs-GO	0.002–10.3	1.26	2	<5 s	Wu et al. (2013)
Pd nanosponges	1–18	32	2	—	Chen et al. (2020)
Gold microelecctrodes	0.5–50	18,502	218	—	Hovancová et al. (2019)
Au@Ni	0.5–10	23.17	15.7	3 s	Gao et al. (2020)

### Nickel-Based NEGS

Like the cobalt-based sensors discussed above, even nickel-based sensors show better current densities for glucose electrooxidation (Liu et al., 2018b; Dai et al., 2018; Shu et al., 2018; Shabbir et al., 2020; Usman et al., 2020). Nickel oxide (NiO) has demonstrated outstanding catalytic activity and excellent stability for the development of glucose sensing devices. Furthermore, these sensors display enhanced properties with short retaliation time, superior sensitivity, lower level of recognition, good recyclability and stability, and a sizeable linear reciprocation window for glucose concentrations (Toghill et al., 2010). However, electrodes based on nickel NPs show low electroanalysis strength because nickel NPs are easily degraded through detection (Toghill and Compton, 2010). Thus, finding a stable structure to increase electrode stability is a necessity (Lee et al., 2018; Mei et al., 2018; Mei et al., 2019; Nurhayati et al., 2020; Li et al., 2021). For instance, NiO@SiNPs based composite materials have also shown promising results towards the electrooxidation of glucose (Naikoo and Din Sheikh, 2019). Nickel oxide electrodes developed by Singer et al. (2020) showed high sensitivity of 4,400 μA mM^−1^ cm^−2^ and a high LOD of 7 nM. Also, the resultant NEGS displayed a wide linear range of 0.5 μM—9 mM with excellent reproducibility and selectivity for glucose against ascorbic and uric acids, serotonin, and dopamine. In another similar study, a NEGS was developed using a platinum electrode functionalized by activated carbon nanotubes @ graphene oxide/nickel hydroxide-Nafion hybrid composite (Mohammadi et al., 2018). This sensor also showed a relatively high sensitivity of 40 nA and a LOD of 0.75 μM. In addition, the sensor was recorded to have high reproducibility, enhanced selectivity, and a rapid response time of less than one second.

**FIGURE 6:**
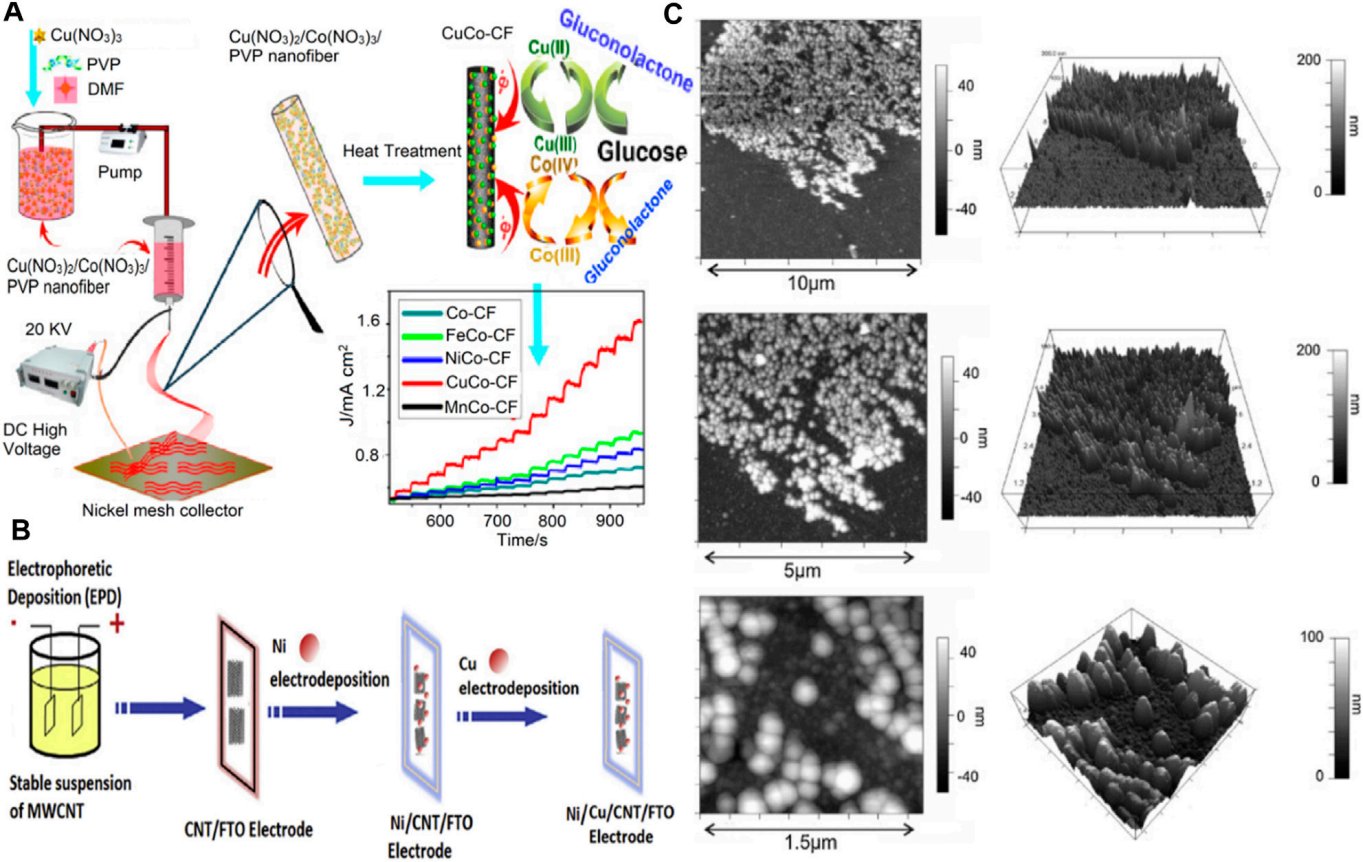
Nickel based NEGS. **(A)** Illustration showing the synthesis routes of CuCo–CFs and the comparison of their catalytic effect to other MCo–CFs. Adapted with permission from ref. (Li et al., 2015), copyright@2015 (Elsevier) **(B)** Schematic representation of nickel/copper/carbon nanotubes nanocomposite electrode. Adapted with permission from ref. (Ammara et al., 2018), copyright@2018 (Elsevier) **(C)** *In situ* EC-AFM images of a nickel modified boron-doped diamond electrode. Nickel was deposited at −1.2 V (vs Ag/AgCl) for 180 s without stirring or degassing, from a 1 mM Ni(NO_3_)_2_ in 0.1 M sodium acetate buffer solution (pH 5). Adapted with permission from ref. (Toghill et al., 2010), copyright@2010 (Elsevier).

Figure 6 discusses these steps of fabrication of nickel based NEGS and their electrodes. It also shows the electrochemical-atomic force microscopy (EC-AFM) and energy dispersive X-ray spectroscopy (EDS) of the nickel modified electrodes used that gives an idea of the surface of the electrodes used.

The potential mechanism of electrocatalysis for NiO nanosheets against glucose is attributed to the redox reaction between glucose molecule and Ni^2+^ ions on the NiO surface under electrochemical conditions as shown below (Tomanin et al., 2018).

$${\text{Ni}}^{2+}{+\text{OH}}^{-}\to {\text{Ni}}^{3+}{+e}^{-}$$

$${\text{Ni}}^{3+}+\text{glucose}\to {\text{Ni}}^{2+}+\text{gluconic acid}\text{.}$$

Firstly, the electrochemical oxidation of Ni^2+^ to Ni^3+^ occurs, followed by electrooxidation of glucose (C_6_H_12_O_6_) to gluconolactone (C_6_H_10_O_6_), then converted to gluconic acid. Eventually, a gluconic acid combines with water producing gluconate and H^+^ ions (Bach et al., 2019).

The linear range, sensitivity, LOD, and response time of various electrode materials based on Ni are explored and discussed in Table 1.

### Zinc-Based NEGS

Zinc oxide (ZnO) nanostructures have strong sensing performance toward the bio-analyte that makes them an excellent candidate to be employed as active sites in electrochemical biosensors (Li et al., 2014). ZnO nanostructures are easily synthesized at low temperatures and demonstrate various morphologies with excellent electrical characteristics, high crystallinity, and strong optical properties (Tripathy et al., 2012; Tripathy et al., 2016). Moreover, ZnO nanostructures offer a wide surface area for modifying nanostructures to obtain valuable NEG sensor devices (Ahmad et al., 2017a; Ahmad et al., 2017b). Multiple investigations on ZnO-based hybrid nanostructures have shown their improved catalytic activity due to their rapid electron transfer and the more excellent surface-to-volume ratio of mixed materials (Perumal et al., 2015; Xie et al., 2016; Xie et al., 2018a). For example, Vinoth et al. (2021) developed zinc oxide quantum dots on carbon nanotubes nanocomposites based glucose sensors that showed a high sensitivity of 9.36 μA μM^−1^ and a LOD of 0.208 μM. The sensor also gave reproducible results within 3 s. In addition, the sensor displayed great selectivity to glucose molecules against ascorbic acid, sucrose, and dopamine. In another study by Haghparas et al. (2021), CuO/ZnO microstructures were developed that exhibited a wide dynamic range of 500 nM to 100 mM and high sensitivity of 1,536.80 μA mM^−1^ cm^−2^. The sensor displayed a LOD of 357.5 nM and gave rapid results in 1.6 s. Likewise, similar observations were recorded in a study by Awais et al. (2021). The authors developed gold-ZnO nanorod based glucose sensors that demonstrated a wide linear range up to 15 mM, a LOD of 0.12 μM, and great sensitivity of 4,416 μA mM^−1^ cm^−2^ The team recorded high reproducibility, selectivity, and stability of this sensor.

The mechanism of action of such a sensor was demonstrated by Dar et al. (2011), Ridhuan et al. (2018). The possible mechanism of glucose oxidation is as follows:

$${\text{O}}_{2}⇌{\text{O}}_{2}\text{ads}\left(\text{ZnO}\right)$$

$${\text{O}}_{2}\text{ads}\left(\text{ZnO}\right){+2\text{e}}^{-}\left(\text{ZnO}\right)⇌{2\text{O}}^{-}\text{ads}\left({\text{O}}^{-}/{{\text{O}}^{-}}_{2}\right)$$

$${\text{Glucose}+\text{O}}^{-}⇌\text{Glucono-δ-lactone}+{2\text{e}}^{-}$$

The linear range, sensitivity, LOD, and response time of various electrode materials based on Zn are explored and discussed in Table 1.

### Copper-Based NEGS

Because of their high surface to volume ratio, copper-based nanomaterials serve as excellent candidates for NEGS development. Their high sensitivity and selectivity give them an upper hand over the other materials. These elements are cheaply available, show enhanced electrochemical features and allowed for easy tuning of the copper oxide structures within the sensor (Liu et al., 2016b). These properties were seen in the work put forward by Ahmad et al. (2017a). They developed highly active sensing electrodes by exploring ZnO nanorods functionalized with CuO, giving stable, selective, and reproducible results towards glucose electrooxidation. In other related studies carried out by Jiang et al. (2017) and Khoshroo et al. (2020) closely similar results were observed. In another study by Amirzadeh et al. (2018), a NEGS was developed based on copper oxide nanoparticles that showed high sensitivity and reproducibility, a broad linear range upto 10 mM and a high sensitivity of 663.2 μA mM^−1^ cm^−2^. Likewise, in a remarkable work put forward by Haghparas et al. (2020), a CuO hollow sphere structure based sensor was developed. This sensor demonstrated a good linear range between 1 μM and 16 mM with a high sensitivity of 35.2 ± 0.4 μA mM^−1^ cm^−2^ and an extremely low limit of detection of 1 μM. In another recent study by the same author a NEGS was developed using CuO and ZnO microstructures. This sensor gave a very wide dynamic range of 500 nM—100 mM, sensitivity of 1,536.80 μA mM^−1^ cm^−2^ and a LOD of 357.5 nM (Haghparas et al., 2021). In addition, the sensor showed a rapid response time of 1.6 s and displayed a prolonged shelf-life, great stability, reproducibility, and high selectivity for glucose molecules. Also, the authors recorded that this sensor could detection glucose molecules in human serum samples.

**FIGURE 7:**
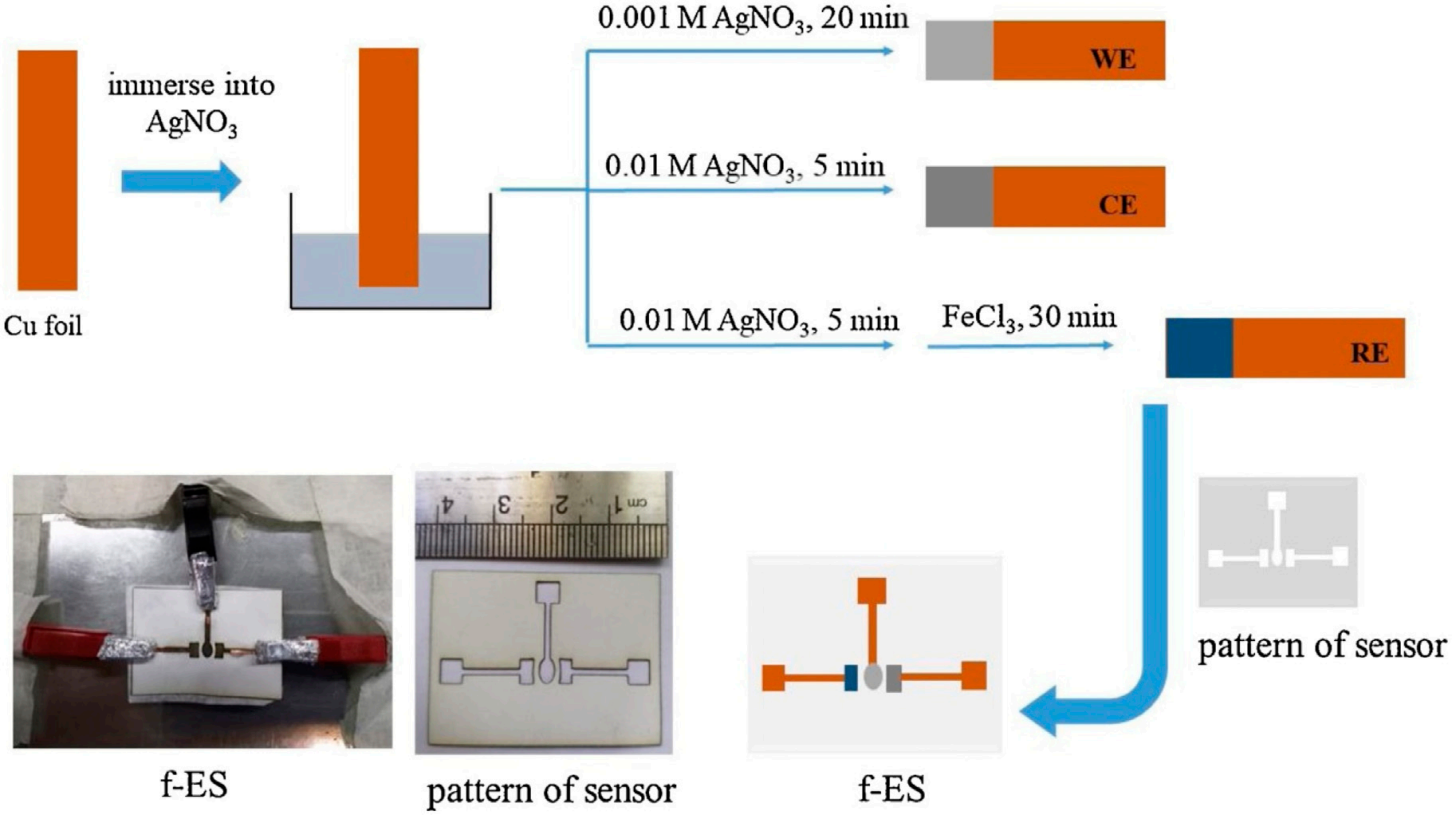
Different steps showing the preparation of the flexible electrochemical NEG sensor (f-ES) on a copper tape platform. Reproduced with permission from ref. (Khoshroo et al., 2020), copyright @ 2020 (Elsevier).

Figure 7 below shows the different steps involved in the preparation of the flexible electrochemical NEG sensor (f-ES) on a copper tape platform.

The plausible mechanism behind the glucose detection by NEGS composed of copper oxide is based on the oxidation of Cu(II) to Cu(III) as described below (Marioli and Kuwana, 1992).

$${\text{CuO}+\text{OH}}^{-}\to {\text{CuOOH}+\text{e}}^{-}$$

$${\text{CuO}+\text{H}}_{2}{\text{O}+2\text{OH}}^{-}\to {\left[\text{Cu}{\left(\text{OH}\right)}_{4}\right]}^{2-}{\;+\text{ e}}^{-}$$

The second step involves deprotonation of glucose, followed by an oxidation step, and eventually hydroxylation.

$$\text{Cu}\left(\text{III}\right)+{\text{glucose}+\text{e}}^{-}\to \text{gluconolactone}+\text{Cu}\left(\text{II}\right)$$

Gluconolactone→gluconic acid

Another glucose oxidation pathway can also occur under alkaline conditions. In this reaction, compounds like formate and carbonate are formed as products. The linear range, sensitivity, LOD, and response time of various electrode materials based on Cu are explored and discussed in Table 1.

### Ferric Oxide-based NEGS

**FIGURE 8:**
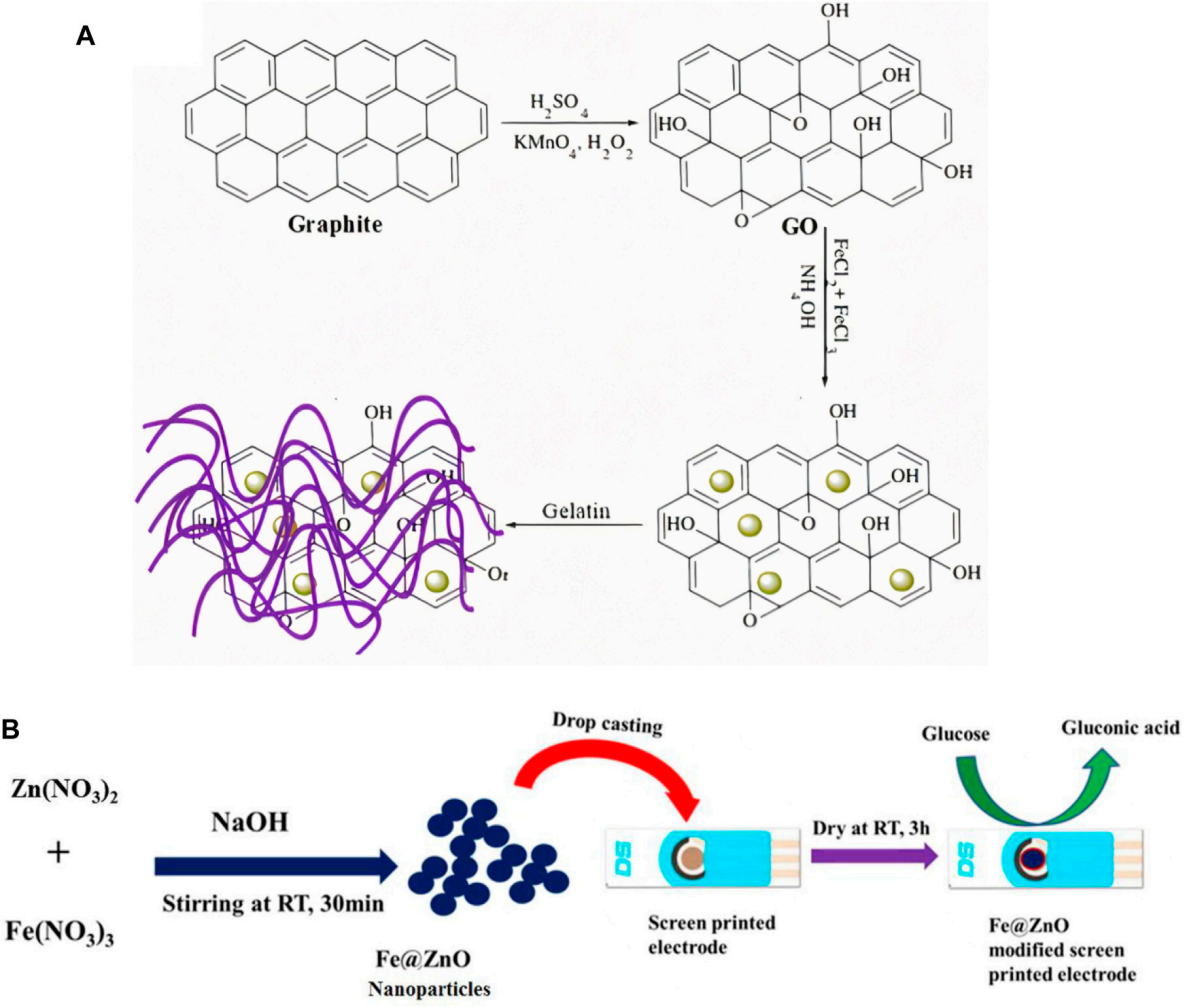
Different steps involved in the fabrication of iron based NEGS **(A)** Schematic representation showing Fe_3_O_4_-rGO-gelatin nanocomposite preparation on GCE sensing electrode. Adapted with permission from ref. (Morteza Naghib et al., 2016), copyright @ 2016 (ESG) **(B)** Schematic diagram showing the synthetic route to Fe@ZnO nanoparticles and fabrication of Fe@ZnO/SPE for glucose oxidation. Adapted with permission from ref. (Raza and Ahmad, 2018), copyright @ 2018 (Elsevier).

Various studies have been done on ferric oxide-based sensors for glucose detection (Cummings et al., 2008; Xia and Ning, 2010; Masoomi-Godarzi et al., 2014; Abdul Amir AL-Mokaram et al., 2016; Morteza Naghib et al., 2016; Zhou et al., 2017) at neutral pH. For instance, Raza and Ahmad (2018) developed a Fe@ZnO based sensor through an annealing process. Figure 8 shows the different steps involved in the fabrication of iron-oxide based NEG sensor. This composite produced was dropped cast on a screen-printed electrode. The glucose detection occurred at a neutral pH of 7.4 and showed a LOD of 0.3 μM. In another study by Abrori et al. (2020), Fe_3_O_4_ based NEGS was developed that could electrochemically detect glucose molecules and displayed a high sensitivity of 4.67 μA mM^−1^ cm^−2^ and a LOD of 15.70 μM. The sensor displayed a high sensitivity and selectivity for glucose molecules.

The reaction occurs at the surface of ferric oxide, and the steps of the reaction for glucose detection include (Masoomi-Godarzi et al., 2014):

$$2\text{Fe}\left(\text{III}\right)+\text{glucose}\to 2\text{ Fe }\left(\text{II}\right)+{\text{gluconolactone}+\text{H}}_{2}\text{O}$$

$${\text{gluconolactone}+\text{H}}_{2}\text{O}\to {2\text{H}}^{+}+\text{gluconate}$$

$$2\text{Fe}\left(\text{II}\right)\to 2\text{Fe}\left(\text{III}\right)+{2\text{e}}^{-}$$

Though the reaction occurs successfully, the sensor’s sensitivity is lower than the sensors that function at a higher pH level (Morteza Naghib et al., 2016). The linear range, sensitivity, LOD, and response time of various electrode materials based on Fe are explored and discussed in Table 1.

### Manganese Oxide-based NEGS

Various studies have been done on manganese oxide-based sensors for glucose detection (Yang and Hu, 2010; Si et al., 2013; Wang et al., 2015; Liu et al., 2016a). However, the lowered conductive properties of manganese oxide (MnO_2_) films make it an unfavorable choice in glucose detection (Yang and Hu, 2010). In this study by Yang et al., such a sensor showed improved sensing of 18.9 μM^−1^cm^−2^. Chen et al. (2008) showed that usage of multiwalled carbon nanotube also improved the conductivity and electrocatalytic capacity of MnO_2_. The immediate interactions between MnO_2_ and glucose molecules occur as (Si et al., 2013):

$${2\text{MnO}}_{2}+{\text{C}}_{6}{\text{H}}_{12}{\text{O}}_{6}\left(\text{glucose}\right)\to {2\text{MnOOH}+\text{C}}_{6}{\text{H}}_{10}{\text{O}}_{6}\left(\text{gluconolactone}\right)$$

Apart from MnO_2_, Mn_3_O_4_ also acts as a supreme catalyst in NEGS and possesses sensitivity of 360 μA mM^−1^cm^−2^ during glucose detection (Zhuang et al., 2010). The linear range, sensitivity, LOD, and response time of various electrode materials based on Mn are explored and discussed in Table 1.

### Titanium Oxide-based NEGS

**FIGURE 9:**
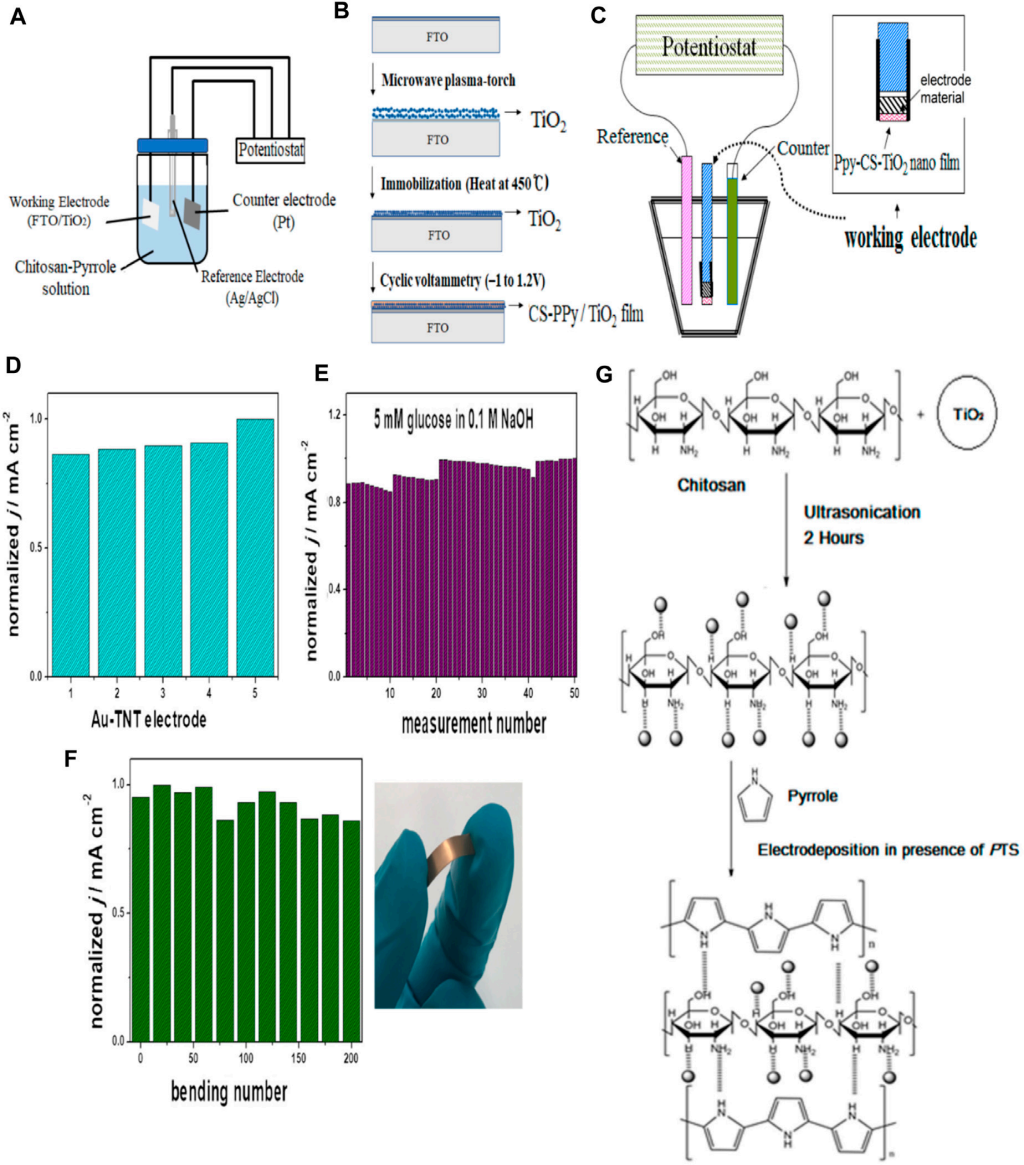
Titanium based NEGS **(A)** Schematic diagram of a three-electrode measuring system **(B)** Schematic diagram showing the preparation of CS-PPy/TiO_2_ nanocomposite films on fluorine-doped tin oxide coated glass slide. Adapted with permission from ref. (Jeong et al., 2021), copyright@2021 (MDPI) **(C)** The electrochemical cell of Ppy-CS-TiO_2_ film preparation. Adapted with permission from ref. (AL-Mokaram et al., 2017), copyright@2017 (MDPI) **(D)** Reproducibility and stability of 50 nm Au-TiO_2_NTs electrode in the case of prolonged measuring **(E)** Reproducibility and mechanical bending of 50 nm Au-TiO_2_NTs electrode **(F)** Reproducibility in 5 mM glucose in 0.1 M NaOH. Photograph shows the maximal deformation of electrode. Adapted with permission from ref. (Grochowska et al., 2019), copyright@2019 (Elsevier) **(G)** The mechanism of Ppy-CS-TiO_2_ film electrodeposition. Adapted with permission from ref. (AL-Mokaram et al., 2017), copyright@2017 (MDPI).

There have been various studies done on titanium oxide-based sensors for glucose detection (Song et al., 2011; AL-Mokaram et al., 2017; Grochowska et al., 2019; Jeong et al., 2021). TiO_2_ acts as a direct photocatalyst in NEGS owing to its enhanced surface to volume ratio (AL-Mokaram et al., 2017). Song et al. (2011) discovered the occurrence of a chemical change between TiO_2_ and TiOOH during the oxidation of glucose molecules. The authors studied that when glucose molecules were subjected to UV light treatment in a Pt/titania nanotube-based sensor, the activity of the TiO_2_ increased and therefore was a great candidate as catalysts in NEGS. During the sensing process, nanocomposite film was deposited on the ITO electrode, forming the CS-Ppy-TiO_2_ nanocomposite film, as shown in Figure 9.

The possible mechanism of glucose oxidation using Ti based oxide occurs *via* the following steps (AL-Mokaram et al., 2017; Yen et al., 2020):

$$2\;{\text{TiO}}_{2}+{\text{C}}_{2}{\text{H}}_{12}{\text{O}}_{6}\left(\text{Glucose}\right)\to 2\;{\text{TiOOH }+\text{C}}_{6}{\text{H}}_{10}{\text{O}}_{6}\left(\text{Gluconolactone}\right)$$

$$2\;\text{Ti}\left(\text{IV}\right)+{\text{C}}_{2}{\text{H}}_{12}{\text{O}}_{6}\left(\text{Glucose}\right)\to 2\text{ Ti}\left(\text{II}\right)+{\text{C}}_{6}{\text{H}}_{10}{\text{O}}_{6}\left(\text{Gluconolactone}\right)+{\text{H}}_{2}{\text{O}}_{2}{\text{C}}_{6}{\text{H}}_{10}{\text{O}}_{6}\left(\text{Gluconolactone}\right)+{\text{H}}_{2}\text{O}\to 2\;{\text{H}}^{+}+{\text{C}}_{6}{\text{H}}_{12}{\text{O}}_{7}\left(\text{Gluconate}\right)$$

$$2\;\text{Ti}\left(\text{II}\right)\to 2\;\text{Ti}\left(\text{IV}\right)+2\;{\text{e}}^{-}$$

The linear range, sensitivity, LOD, and response time of various electrode materials based on Ti are explored and discussed in Table 1.

### Iridium Oxide-based NEGS

There have been various studies done on iridium oxide-based sensors for glucose detection (Dong et al., 2018a; Dong et al., 2019). NEGS are made using this composite *via* electro spin annealing method and show enhanced function under an alkaline environment. Dong et al. (2018a) studied IrO_2_ nanofibers for glucose oxidation and concluded that due to the distinctive crystallinity of this nanofiber, it showed good electrocatalytic activity with a sensitivity of 22.22 μA mM^−1^cm^−2^. The LOD of the sensor was noted to be 2.9 μM. However, this catalyst shows lower sensitivity than other metal oxide-based nanomaterials, like cobalt oxide-based sensors. Therefore, the sensitivity of IrO_2_ can be enhanced by doping electrospun nanoclusters with gold, as shown by Dong et al. (2019).

Glucose oxidation using IrO_2_ has not been extensively studied. Ir is often linked to or supported with Ni structures, like IrO2@NiO core-sheath structure for the growth of Ni metal oxides that are further used for glucose sensing purposes (Wang et al., 2016b). The glucose oxidation on IrO_2_ based sensor occurs in an alkaline solution and is a two-step process (Dong et al., 2018a):

$${\text{IrO}}_{2}+{2\text{OH}}^{-}\to {\text{IrO}}_{2}{\left(\text{OH}\right)}_{2}+{2\text{e}}^{-}$$

$${\text{IrO}}_{2}{\left(\text{OH}\right)}_{2}+2\;\text{glucose}\to {\text{IrO}}_{2}+2\;{\text{gluconolactone}+2\text{H}}_{2}\text{O}$$

The linear range, sensitivity, LOD, and response time of various electrode materials based on Ir are explored and discussed in Table 1.

### Rhodium Oxide-based NEGS

There have been various studies done on rhodium oxide-based sensors for glucose detection (Dong et al., 2018b). Dong et al. (2018b) successfully showed that rhodium oxide nanocorals (Rh_2_O_3_ NCs) modified glass carbon electrode can be used during electrochemical glucose oxidation. This sensor showed a high sensitivity of 11.46 μA mM^−1^ cm^−2^ and a LOD of 3.1 μM. The authors developed this sensor based on the study by Ding et al. (2010) who gave a two-step synthesis pathway for Co_3_O_4_ nanofibers that were cast on the surface of a glass carbon electrode and bound to Nafion. This Co_3_O_4_ NFs-Nafion/GCE based NEGS was also shown to perform well during electrooxidation of glucose in an alkaline environment.

Dong et al. (2018b) chose to predict the correlation between rhodium and cobalt because of the proximity of these two elements on the periodic table. Being from the same group, these elements show similar metal oxide property and thus similar catalytic activity during glucose oxidation. The mechanism of glucose oxidation on rhodium based sensors can be observed from the world put forward by this work. Dong et al. proposed the two-step mechanism of glucose oxidation on Rh_2_O_3_ nanoclusters in alkaline solution.

$${\text{Rh}}_{2}{\text{O}}_{3}+{2\text{OH}}^{-}+{\text{H}}_{2}\text{O}\to 2\text{RhO}{\left(\text{OH}\right)}_{2}+{2\text{e}}^{-}$$

$$2\text{RhO}{\left(\text{OH}\right)}_{2}+2\text{glucose}\to {\text{Rh}}_{2}{\text{O}}_{3}+{2\text{glucolactone}+3\text{H}}_{2}\text{O}$$

The linear range, sensitivity, LOD, and response time of various electrode materials based on Rh are explored and discussed in Table 1.

### Platinum Oxide-based NEGS

**FIGURE 10:**
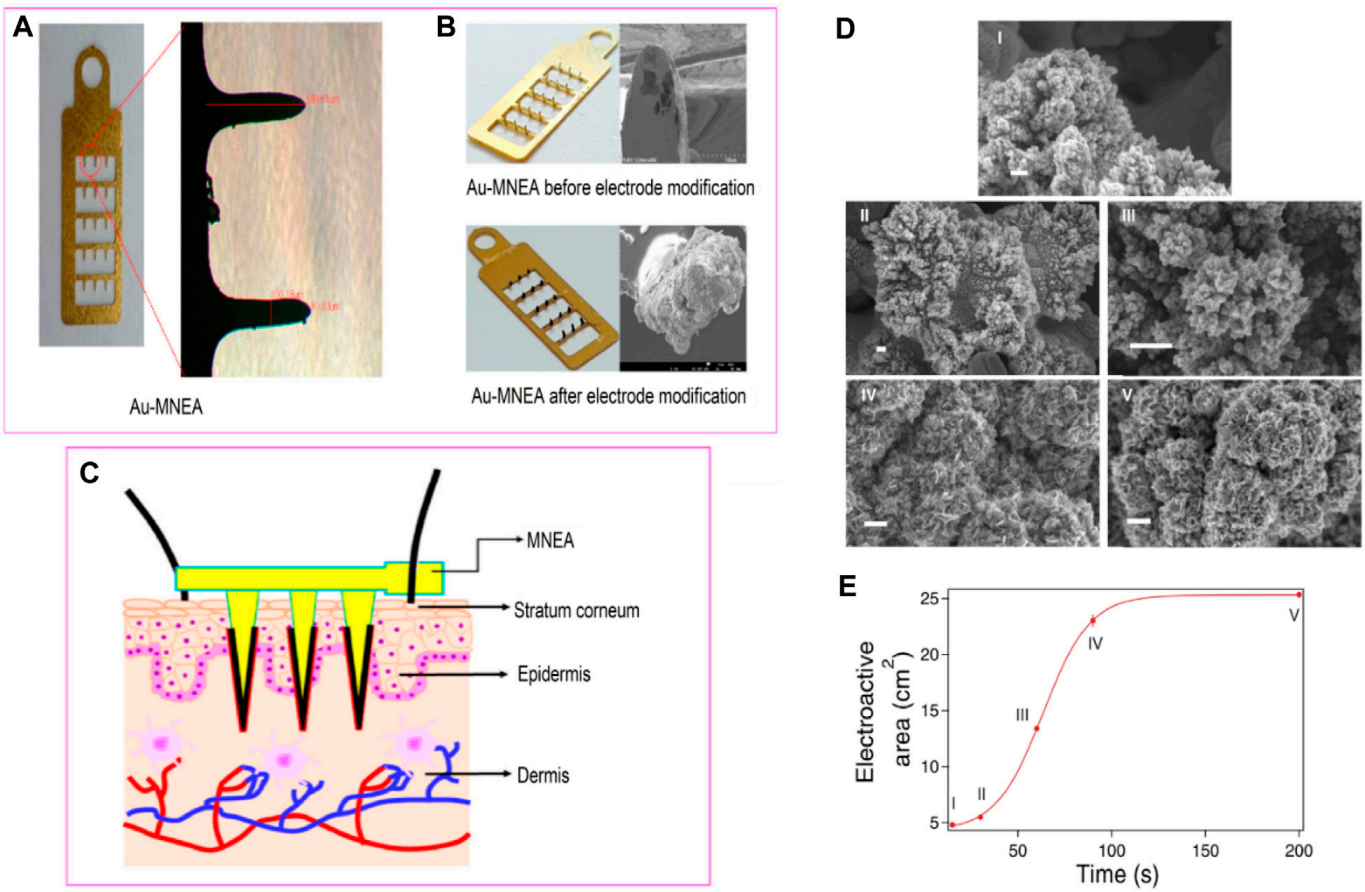
**(A)** Optical micrographs of bare Au-modified microneedle electrode array (MNEA) showing a length of 599.61 µm and width of 100.16 µM. **(B)** Optical and SEM Micrographs of the fabricated MNEAs before and after the catalytic Pt-black layer deposition. **(C)** The schematic illustration of MNEA insertion into superficial dermis of rat skin. Adapted with permission from ref. (Chinnadayyala and Cho, 2021), copyright@2020 (MDPI). **(D)** SEM images of Pt coatings obtained at different deposition times (bars: 200 nm). **(E)** Evolution of the electroactive area with deposition time. Adapted with permission from ref. (Taurino et al., 2015), copyright@2015 (Nature).

Pt nanomaterials show enhanced performance in glucose electrochemical detection, and many recent studies have supported this view (Sakr et al., 2020). In addition, these structures do not require complicated steps and are often produced in a single step reaction (Taurino et al., 2015; Figure 10). Pt-based biosensors demonstrate high reproducibility, stability, and sensitivity towards glucose detection. Wu et al. (2013) developed such a NEGS that displayed a rapid response time of fewer than 5 s and a wide linear range of 2 μM–10.3 mM with a high sensitivity of 1.26 μA mM^−1^ cm^−2^ and a LOD of 2 μM that is relatively low compared to other metal oxides NEGS. Many recent studies have stressed the high stability of Pt-based electrodes that increase the functionality and overall stability of such a sensor (Unmüssig et al., 2018). In addition, such modified sensors have been reported to display 10,000 times increased sensitivity under physiological pH conditions compared to the other sensors (Unmüssig et al., 2018).

### Palladium Based NEGS

Waqas et al. recently developed palladium-based sensors. The employed mixed metal alloy nanoparticles that included Pd, Mn, and rGO and could detect glucose molecules under alkaline conditions. The hybrid sensor demonstrated superior electrochemical functioning during the sensing and enhanced sensitivity and selectivity towards glucose molecules (Waqas et al., 2020). In another similar study, excellent properties of palladium were studied that also showed high reproducibility and selectivity for glucose molecules during the detection (Promsuwan et al., 2019). In another research by Chen *et al.*, unique Pd nanosponge architectures were developed that showed a broad linear range of 1—18 mM and high sensitivity of 32 μA mM^−1^ cm^−2^ (Chen et al., 2020). In addition, the sensor was found to be highly stable for long durations and displayed a LOD of 2 μM during glucose sensing.

### Gold-Based NEGS

Gold-based NEGS has also been shown to possess high stability, reproducibility, and selectivity for glucose molecules. The biggest advantage of these gold-based sensors is that they remain unaffected by changes in pH, temperature, and other chemicals in the vicinity (Gao et al., 2020). A novel and stable NEGS based on gold nanoclusters was developed by Hovancová et al. (2019). This sensor demonstrated a high sensitivity of 185.2 mA mM^−1^cm^−2^ and a linear range of 0.5–50 mM with a LOD of 218 μM. In addition, the authors also noted that these gold microelectrodes hold promise for the miniaturisation of the glucose-sensing systems. In another similar work, NEGS were developed based on gold-nickel nanoparticles and high sensitivity of 23.17 μA cm^−2^ mM^−1^ was observed along with a LOD of 0.0157 mM in under 3 s (Gao et al., 2020). In addition, this sensor was undisturbed by toxic chemicals like chloride molecules and thus remained active for long durations. Similarly, in another recent study by Chen et al. (2021), closely similar observations were recorded. This sensor was based on gold nanoparticle-modified indium tin oxide electrode and boronate affinity and displayed a wide linear range of 0.5–30 mmol/L and a LOD of 43 μmol/L. This sensor showed a high functionality under physiological pH and was also found to be biocompatible. This gold-based are promising candidates for the development of enzyme-free glucose sensors.

## Challenges and Possible Solutions for the Development of NEGS

The challenges observed in the development of NEGS starts from the large number of production steps involved. These include the tedious process of cleaning electrodes, selecting the binders, and their respective usage. In addition, the preparation of electrode materials for NEGS and their loading activity results in increased time consumption and overall expenses. Moreover, the NEGS selectivity is affected by the enhanced contact resistance observed in catalysts and the current collector. Furthermore, the byproducts of glucose oxidation sometimes get attached to the surface of electrodes in NEGS, which affects the sensitivity (Rong et al., 2007). Challenges like the robustness of the NEGS also pose a significant concern. The other bottlenecks of NEGS include the possibility of low stabilization due to interference from oxidizable molecules like uric acids. And increased poisoning of electrode materials due to chloride ions in the actual serum or blood samples is one of the most significant drawbacks of NEG sensors. More importantly, as discussed in the previous section, these sensors show maximum activity under alkaline conditions, thus posing a substantial concern to detect glucose under the physiological pH range. Hence, their applicability in clinical settings is a significant concern that needs our attention.

Despite the numerous studies in recent years related to NEGS, more research needs to be done to enhance its design and development for the promising increase in selectivity, sensitivity, stability, response time, and affordability. The selectivity of NEGS can be increased by using sensors devoid of binders and electrodes based on nanofiber, gel, or foil-based membranes. Eventually, the issue due to chloride ion poisoning can be overcome by exploring advanced materials for the design and development of NEGS and making the sensor more resistant and better preserved. The composite materials such as active carbon and graphene combined with metal nanostructures and metal oxides can be potentially promising candidates for NEGS development (Gnana Kumar et al., 2017). In addition, as discussed previously, Pt, Pd, and Au based sensors function under the physiological pH range and thus can detect glucose molecules directly from blood samples. Hence, such sensors can employ noble metals or are functionalized with such elements to increase their functionality in the neutral pH range. In addition, pH can be regulated by attaching a bioelectronic pH control to the sensor, as Strakosas et al. (2019) successfully did in their studies. This change will help improve the working of the sensor to allow glucose sensing in biological fluids.

## Comparison of EGS With NEGS

Apart from the sensors, many methods are available to detect and quantify glucose levels in a given sample. Such methods include capillary zone electrophoresis (Sastre Toraño et al., 2019), Fourier transform spectroscopy (FTIR) (Petibois et al., 1999), high-performance liquid chromatography (HPLC) (Gika et al., 2016) among others. These methods, however, are comparatively more expensive and tedious because of the requirement of further steps during the detection process, laboratory professionals, and the inability to be developed into sophisticated sensing devices which has made electrochemical sensing devices better choices rather than cumbersome traditional methods. These detection systems show high sensitivity and selectivity. Additionally, they allow for a compact design that can be easily used in glucose diagnosis without trained professionals or expensive devices. These glucose sensors are either enzyme-based glucose sensors (EGS) or NEGS. The EGS requires an intermediary enzyme (glucose oxidase or glucose dehydrogenase) to detect and quantify the glucose samples accurately. On the contrary, NEGS do not necessarily need an intermediary; instead, they make the direct use of glucose available in the sample to quantify their levels.

EGS suffer from the consequences of enzyme denaturation, inefficiency in the transfer of electrons within electrode surface and enzymes, inconvenient immobilization techniques, inability to reproduce results, deformation due to heat and other external chemical molecules in the vicinity of the samples. NEGS plays a crucial role in solving these challenges. NEGS are comparatively cheaper than EGS. The lack of enzymes confer them better stability and leaves them unaffected by external conditions like pH, temperature, ionic strength (Wang, 2008; Popov et al., 2021). Moreover, their ability to give quick results with high sensitivity gives NEGS an upper hand on EGS. However, as discussed earlier, conditions like instability, activity loss, and surface poisoning may occur on rare occasions because of faulty or old electrodes in NEGS.

## Advantages and Disadvantages of NEGS

Metal oxides have gained widespread popularity in recent decades because of their unusual electrocatalytic activity and are used in many electrochemical devices (Hassan et al., 2021). As discussed before, metal oxides, including Co, Ni, Zn, Cu, Fe, Mn, Ti, Rh, Ir, Pt, Pd, and Au have proven to be promising elements for glucose oxidation in glucose sensing devices. These materials are easily, quickly, abundantly, and cheaply available in nature and are eco-friendly (Vennila et al., 2017). They also show enhanced catalytic activity (Fan et al., 2019). Moreover, sensors with two or more of these metals in combination also display excellent sensing results. Compared to traditional sensors that employ enzymes, metal oxide sensors do not undergo any enzymatic degradation and thus remain active for prolonged durations. For instance, Ramachandran et al. (2016) showed in their studies that NEGS developed using Ni-Co nanowires exhibited high sensitivity and selectivity towards glucose detection. In another similar research carried out by Suneesh et al. (2015), a NEGS based on Co-Cu alloy NPs also served as an excellent sensing device for quantifying glucose levels. In addition, NEGS has proven to exhibit better linear range and lowered potential at which it operates than EGS.

Despite the several advantages of metal oxide-based NEGS, there are a few shortcomings of NEGS. For instance, highly conductive carbon-based nanomaterials are the best choices for the electrooxidation of glucose; however, their stability is a big concern. Therefore, researchers have determined that metal oxides such as Co, Ni, Zn, Cu, Ti, Mn, Ti, Ir, Rh and their bimetallic nanomaterials have promising potential to foster and promote NEGS in mass production. However, a few metal-based NEGS have shown lowered selectivity at high voltages (Gao et al., 2020). There is no sophisticated control of protective sheath, thickness, and pore size of the nanoporous layer to allow NEGS to bear the capacity to work on plasma, human serum, and blood when undiluted. Furthermore, disturbances arising from different electro-active and electro-inactive chemical species still needs adjustments (Hwang et al., 2018).

## Conclusion and Future Prospective

Rapid progress in nanoscience and nanotechnology has fueled the diversification and sophistication of NEGS development over the past decade because of the alarming increase in diabetes worldwide. The significant advances in medical applications for enzyme-free systems using nanoporous materials as potential electrodes are the most distinguished outcome (Park et al., 2012). With the increasing number of diabetic cases worldwide, there is an urgent need to design and develop highly advanced NEGS capable of giving highly selective and specific results. Despite the recent progress made in NEGS, there are still many shortcomings that still need to be adequately addressed. Further research needs to be carried out to understand better the effects of the host matrix’s shape and structure during glucose detection and their interactions with each other. An in-depth analysis of changeable pore size and properties can develop a more significant number of active sites and better surface area, increasing the NEGS efficiency. Atoms-based, molecules-based, and electronic-based models can create a better protocol for the experiments using NEGS. This is also important to further understand the detailed mechanisms during glucose oxidation and their relation with the sensors. Improvement of NEG sensors for glucose detection has attracted scientists over the past couple of decades. Researchers have paid ample attention to metal-based electrodes as an alternative to electrodes based on noble metals to fabricate reliable glucose sensors. They showed promising potential in glucose sensing applications because of their high catalytic performance, selectivity, and sensitivity. However, noble metals being more versatile and retain their activity with changes in external environmental conditions (like temperature and pH), are better preferred for developing NEGS that can function under physiological range. Thus, metal-oxides functionalized with noble metals and their alloys or hybrid electrode structures can be used to develop NEGS to detect glucose under clinical settings.

This review has briefly outlined the direct electrochemical oxidation of glucose as an excellent technique for glucose detection. Moreover, the oxidation mechanism applied to detect glucose by using Co, Ni, Zn, Cu, Fe, Mn, Ti, Rh, Ir, Pt, Pd, Au nanomaterials has been detailed. Substantial findings have exposed that the sensors based on the materials mentioned earlier offer greater efficiency and could be a promising potential candidate for developing glucometer devices. To overcome the growing health apprehension because of the snowballing number of diabetics, it is of the utmost concern for researchers to develop efficient and reliable glucose sensors for the early detection of diabetic patients. Fabrication with metal oxide nanostructures will result in a combination of excellent properties and provide a novel approach for sensitive NEG sensors. Such efforts will mostly make the processes of diagnosis easier, quicker, and less invasive. Personalised medicine is also gaining interest, and it is predicted that overall results will empower the nanotechnology market. Although metal electrodes are appealing sensing candidates, further professional, academic and technological research is required for miniaturization and commercialization.
